# Supplementary material for: Calibrated-Two Optional Randomized Response Techniques (C-TORRT) for the estimation of quantitative sensitive variable information
Source: PLoS One. 2026 Jan 12;21(1):e0339271. doi: 10.1371/journal.pone.0339271 (PMC12795395; doi:10.1371/journal.pone.0339271)
Supplement: S1 File — (DOCX) [file pone.0339271.s001.docx]

**Table A1: CGPA AND NUMBER OF TIMES STUDENTS SEEK ACADEMIC HELP (NTSH)**

| CGPA (Y) | | NTSH (X) | |
| --- | --- | --- | --- |
| 3.81 | 4.55 | 1 | 1 |
| 3.07 | 2.32 | 2 | 1 |
| 3 | 2.9 | 0 | 1 |
| 3.8 | 3.8 | 1 | 2 |
| 3.5 | 3.5 | 1 | 1 |
| 4.3 | 4.54 | 2 | 1 |
| 3.4 | 2.9 | 1 | 1 |
| 3.2 | 2.51 | 2 | 1 |
| 2.4 | 3.08 | 1 | 1 |
| 3 | 3.11 | 0 | 2 |
| 2.59 | 3.48 | 1 | 0 |
| 3 | 3.1 | 2 | 2 |
| 4.2 | 4.1 | 1 | 1 |
| 4.31 | 4.32 | 1 | 2 |
| 3.06 | 3.4 | 1 | 1 |
| 4.06 | 3.8 | 2 | 1 |
| 3.9 | 4.32 | 2 | 1 |
| 3.21 | 3.28 | 0 | 1 |
| 2.53 | 4.4 | 1 | 1 |
| 3.7 | 3.18 | 1 | 2 |
| 4.51 | 3.15 | 1 | 2 |
| 3.29 | 2.98 | 1 | 1 |
| 4.08 | 3.87 | 2 | 2 |
| 4.52 | 3.59 | 2 | 2 |
| 2.5 | 3.9 | 2 | 1 |
| 3.92 | 3.84 | 2 | 2 |
| 3.98 |  | 2 |  |
| 2.56 |  | 0 |  |
| 3.92 |  | 1 |  |
