# Supplementary material for: Calibrated-Two Optional Randomized Response Techniques (C-TORRT) for the estimation of quantitative sensitive variable information
Source: PLoS One. 2026 Jan 12;21(1):e0339271. doi: 10.1371/journal.pone.0339271 (PMC12795395; doi:10.1371/journal.pone.0339271)
Supplement: Attachment — Submitted filename: Response to Reviewers Plos ONE.docx [file pone.0339271.s003.docx]

PONE-D-25-03382

Calibrated-Two Optional Randomized Response Techniques (C-TORRT) for the Estimation of Quantitative Sensitive Variable Information

Reviewer #1:

| S/N | REVIEWER’S COMMENTS | AUTHORS’ RESPONSES |
| --- | --- | --- |
| 1 | Authors are suggested to rewrite (clear and structured) the Abstract which will improve the accessibility of the paper and help readers grasp its core contributions quickly. | DONE |
| 2 | The authors should carefully revise the manuscript to eliminate any unnecessary repetition. | DONE |
| 3 | Provide practical examples of applications. | DONE. SEE PAGE 26-28 |
| 4 | Recheck the manuscript for various grammatical and typo errors. | DONE |

Reviewer #2:

| **S/N** | **REVIEWER’S COMMENTS** | **AUTHORS’ RESPONSES** |
| --- | --- | --- |
| 1 | Abstract should rewrite as it should be indicated the main findings (with some values). | DONE |
| 2 | Abstract should not contain any references | DONE |
| 3 | Reference citation is irregular. It should be in sequence. | DONE |
| 4 | The first paragraph of introduction part is without any reference. | DONE |
| 5 | Equations are not cited properly. Give the reference for each equation. | CORRECTIONS ON EQUATIONS HAVE BEEN DONE |
| 6 | The entire work shows without single results (in terms of graphical representation). | SINCE GRAPHS OF THE RESULTS WILL NOT GIVE DIFFERENT RESULTS, THEY ARE EXEMPTED TO MINIMIZE THE PAGE NUMBER OF THE MANUSCRIPT |
| 7 | . In some of the sentence, it is difficult to understand the meaning of text. | CORRECTED |
| 8 | There is no comparative study with existing work. | THE PROPOSED CALIBRATED RRT MODELS WERE COMPARED WITH THAT OF Gjestvang and Singh (2009) $Z_{GS}$ AND Azeem et al (2023) $Z_{AZ}$. See tables 2a-7 |
| 9 | The data and results are not sufficient. Author must add more results otherwise work will not recommend for publication | DONE. SEE TABLE 5-7 |
| 10 | Result explanation is not sufficient and justified. Give proper reason for all the results and justify. | DONE |
| 11 | Conclusion should have some resulting details with numerical data. | DONE |
